# Visual Agreement Analyses of Traditional Chinese Medicine: A Multiple-Dimensional Scaling Approach

**DOI:** 10.1155/2012/516473

**Published:** 2012-09-17

**Authors:** Lun-Chien Lo, John Y. Chiang, Tsung-Lin Cheng, Pei-Shuan Shieh

**Affiliations:** ^1^Department of Traditional Chinese Medicine, Changhua Christian Hospital, Changhua 50006, Taiwan; ^2^Graduate Institute of Statistics and Information Science, National Changhua University of Education, Changhua 50058, Taiwan; ^3^Department of Computer Science and Engineering, National Sun Yat-Sen University, Kaohsiung 80424, Taiwan

## Abstract

The study of TCM agreement in terms of a powerful statistical tool becomes critical in providing objective evaluations. Several previous studies have conducted on the issue of consistency of TCM, and the results have indicated that agreements are low. Traditional agreement measures only provide a single value which is not sufficient to justify if the agreement among several raters is strong or not. In light of this observation, a novel visual agreement analysis for TCM via multiple dimensional scaling (MDS) is proposed in this study. If there are clusters present in the raters in a latent manner, MDS can prove itself as an effective distinguisher. In this study, a group of doctors, consisting of 11 experienced TCM practitioners having clinical experience ranging from 3 to 15 years with a mean of 5.5 years from the Chinese Medicine Department at Changhua Christian Hospital (CCH) in Taiwan were asked to diagnose a total of fifteen tongue images, the Eight Principles derived from the TCM theorem. The results of statistical analysis show that, if there are clusters present in the raters in a latent manner, MDS can prove itself as an effective distinguisher.

## 1. Introduction

Reliability is an indispensable requirement in the biomedical diagnostics. The intraclass or interclass reliabilities have been proposed by many authors [1–7]. There are many works studying agreement measures for western medical diagnostics. However, only a few of them perform agreement analysis for TCM practitioners. In most of the literature concerning TCM agreement, even though complex combinations of TCM diagnostics are considered, a so-called proportion of agreement measure is adopted. The “proportion of agreement,” shown by evidence, overlooks the possible bias caused by randomness. In order to remedy the bias, Cohen proposed his renowned “alpha” measure. Soon after his contribution, weighted kappa, Fleiss kappa, and so forth had been proposed to deal with more complex data types and more raters. Reference [8] considered a reliability measure called “Krippendorff's alpha” to investigate the agreement of tongue diagnoses when there are many practitioners, and the data is ordinal. Krippendorff's alpha coefficient equal to 0.7343 was reported in their study. 

The core of diagnosis in Chinese Medicine is “pattern identification/syndrome differentiation and treatment” with inspection, listening, and smelling examination, inquiry, and palpation as the bases. Inspection tops the four diagnoses, and tongue diagnosis is a crucial part during observation. The tongue is connected to the internal organs through meridians; thus the conditions of organs, qi, blood, and body fluids as well as the degree and progression of disease are all reflected on the tongue. Organ conditions, properties, and variations of pathogens can be revealed through observation of tongue. Tongue inspection refers to the shape, color, and coating of a tongue that is, the degree of dimension for tongue diagnosis is three. Krippendorff's alpha is a good approach for agreement analysis when evaluating the agreement of many TCM practitioners with ordinal data. However, it is complex, and only a single index representing agreement is rendered. More importantly, Krippendorff's alpha cannot deal with high-dimensional ordinal data obtained through the TCM tongue diagnosis. These two aforementioned pitfalls invalidate the application of Krippendorff's alpha to the analysis of multidimensional agreement data. Other effective means has to be sought.

 In light of the previous observation, we aim at proposing an effective approach to simultaneously deal with highdimensional ordinal data as well as the case when clusters present in the rating result.

A single value of agreement can only represent the “averaging mass” of agreement. We can hardly derive any meaning information out of the single agreement measure, especially when there are clusters present. For example, in the diagnosis of tongue shapes (thick, medium, and thin), suppose that there are three TCM practitioners judging some patients as “thick” and the other three practitioners “medium.” We might reach a low-agreement conclusion, though the agreement is strong, respectively, within each of the two groups. It is interesting that, although it might be low in overall agreement, different TCM prescriptions could work well equally. With these perspectives, an alternative approach, such as multiple-dimensional scaling (MDS), may prove itself as a better alternative to analyzing the agreement of diagnostics among many TCM practitioners with high-dimensional ordinal data. Kupper and Hafner proposed a method to assess the extent of interrater agreement when each unit to be rated is characterized by a subset of distinct nominal attributes [9]. When the attribute data is high-dimensional, the interrater agreement can be treated as the similarity used in multiple-dimensional scaling [10] (MDS). The essence of MDS is an attempt to represent the observed similarities or dissimilarities in the form of a geometrical model by embedding the stimuli of interest in some coordinate space so that a specified measure of distance, for example, Euclidean distance, between the points in the space represents the observed proximities. In other words, MDS is the search for a low-dimensional space where each space point represents stimulus and the distance between points corresponds to dissimilarity.

In this study, we recruited eleven TCM practitioners with ages ranging from 29 to 47. A total of 15 tongue pictures, taken by the Automatic Tongue Diagnosis System (ATDS) developed to extract tongue features to assist clinical diagnosis, are randomly chosen.

For each of these fifteen tongue images, the recruited TCM practitioners have to identify the patterns according to Eight principles. The Eight principal syndromes are made up of four pairs of opposites, namely, Yin and Yang, Cold and Hot, Empty and Full (or Deficiency and Excess), Exterior and Interior. A symptom or disease can possess several of these properties simultaneously. 

## 2. Method and Results

### 2.1. Patients and TCM Tongue Inspectors

Fifteen pictures of tongues are randomly selected from the archive of the Department of TCM, Changhua Christian Hospital (CCH). The pictures were taken by a digital image capturing and analyzing system called ATDS and were rated by eleven TCM practitioners with ages ranging from 29 to 47. The recruited TCM physicians have to classify each image, based on the Eight Principles, according to the features revealed by the tongues.

### 2.2. Statistical Analysis

**Figure 1:**
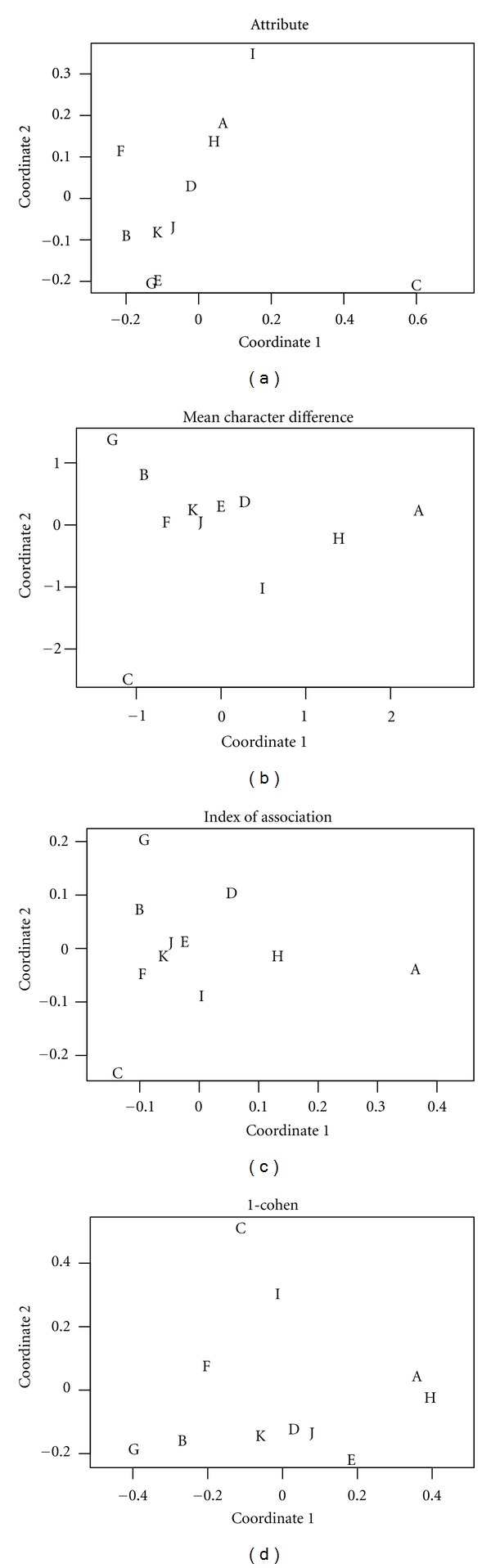
MDS graphs for multiple attributes of Eight Principles for 11 TCM practitioners and 15 patients.

In this study we use four dissimilarity measures to conduct a nonmetric MDS which was first proposed by Kruskal [11, 12]. The four measures refer to Kupper and Hafner's IAMA [9] (interrater agreement for multiple attributes), mean character difference (MCD), index of association [10] (IOA), and average Cohen's kappa (Cohen's kappa). The IAMA measure is a chance-corrected concordance. Among these four measures, IAMA and Cohen's kappa belong to similarity measures, while the other two measure dissimilarity. These four measures will be described in detail in the Appendix.  Table 1 is a summary of the patterns of the fifteen patients that are identified by the eleven TCM physicians of CCH according to the Eight Principles. The letters in the body of the table refer to specific TCM physicians. In Table 2, the dissimilarities obtained by IAMA among the TCM physicians are listed. For example, the interrater agreement between rater A and rater C is 0.2462 therefore the dissimilarity can be defined by 1 − 0.2462 = 0.7538. Naturally, the diagonal entries are identically zero. The MDS graphs of agreement measures by the proposed four approaches are illustrated in Figure 1. The upper-left graph uses IAMA measure to conduct MDS, the upper-right one corresponds to the MCD method, the lower-left one represents the IOA method, and the lower-right one employs averaging Cohen's kappa of each attribute in the eight patterns between two distinct raters. 

## 3. Results

We summarize the diagnoses of the patterns of the fifteen patients in Table 1. According to the four measures mentioned previously, MDS analysis may be conducted to further derive these similarity or dissimilarity measures. Figure 1 shows that the MDS graphs by IAMA and Cohen's kappa are similar. Rater C is an outlier for all these four graphs. Besides, the graphs by IAMA and Cohen's kappa share some characteristics in common. Note raters I and F are a little away from the biggest cluster formed by raters B, D, E, G, J, and K. Secondly, raters A and H form a small cluster. Traditional MDS distances using MCD or IOA lead to similar results. From Figure 1, raters C, I, H, and A are isolated singletons. There exists only one cluster formed by raters B, D, E, F, G, J, and K. In all these four graphs, raters B, D, E, G, J and K form a cluster.

## 4. Conclusion 

In the TCM diagnostics, the practitioners are routinely confronted with a multiple-dimensional qualitative problem of symptom identification. Conventionally, the diagnosis according to Eight Principles summarizes the dynamics of a patient pursuing TCM treatment. When a TCM practitioner receives the information taken by way of the four diagnostics called “inspection, listening (smelling), inquiring and palpation,” he has to distinguish the patterns which are coherent with the symptoms exhibited by the patients. Therefore, how to measure the agreement of the diagnoses according to the vector attributes observed by TCM practitioners is an important issue. 

For a single attribute, the researchers are used to adopt Cohen's kappa, Fleiss kappa, or Krippendorff's alpha to obtain a single-valued agreement measure. There is a drawback in these popular agreement measures. It does not have a rule of thumb to judge the level of agreement. In this study, we introduce a novel approach in deriving interrater agreement including IAMA proposed by Kupper and Hafner and the averaging Cohen's kappa, to calculate dissimilarities between any pair of raters. Using the dissimilarity measures, the MDS analysis can be conducted and an agreement graph is subsequently obtained. Figure 1 shows that rater C remains an outlier for all of the four methods. It might be due to that his diagnosis includes many “mixture” patterns, for example, “Yin” mixed with “Yang,” or “Cold” mixed with “Hot,” and so forth. Rater C is a senior TCM physician in the department of TCM of CCH and has a very long experience of research. Moreover, raters A and H are not only TCM practitioners in CCH, but also participate actively in advanced TCM studies for many years. From these analyses, other than agreement, we can distinguish the raters by clusters. As we mentioned in the Introduction section, the conventional single agreement is quite restricted in terms of successfully interpreting the meaning hidden underneath. It cannot judge whether a given “moderate” agreement coefficient is sufficient to quantify the reliability of TCM diagnostics or not. If there are clusters present in the raters in a latent manner, MDS can prove itself as an effective distinguisher. 

## Figures and Tables

**Table 1:** A summary of the patterns of the fifteen patients identified by the eleven TCM physicians. Alphabets in the table entries correspond to different TCM physicians participated.

				Pattern			
Patient	Yin	Yang	Cold	Hot	Empty	Full	Hematoma
1	BCDFGIJK	CE	BFGIJK	ACEHK	BCDFGIK	ACEFGHJ	ABCDEFGHIJ
2	ABCDEFHIJK		ABCEFHIJK		ABCDFGHIK	EJ	ACDEFGHIJK
3	ABDEFGJK	CHI	BFGIJK	CDEFHIK	ACDFIJK	BCEFGHK	DEGHJ
4	BCEFHIJK	C	ABDEFHIJK	CGK	ACDEFGHJ	BCIK	BCDEFGHIJK
5	ABCEFHIJK	C	ABDEFHIJK	C	ACDFHIK	BCEGJK	BCDEFGHIJK
6	ABCDEFHIJK		ABCDEFHIJK		ABCDEFHIJK	BGK	ABCDEFGHIJK
7	BCEFGHJK	I	ABCDEFHIJK		ABCDEFGHIJK		ABCFGI
8	BCEFHK	CIJ	BDEFGHK	CDIJ	ABCDEHIJK	C	BCEFGHIJ
9	BCDEFGHIJK		BCDEFGHIJK	K	ABCDEFGHIJK	BFG	BCDEFGHIJ
10	CEFGIK	CDJ	EFK	CDFIJ	ACHI	CDEFGJK	ACDEFGHIJK
11	C	ABCDEFGHIJK		ABCDEFGHIJK	CK	BCDEFGHIJ	BCDEFHJ
12	ACFHI	BCEJK	FGI	BCDEFGJK	CG	BCDEFGIJK	ABCEFGHIJK
13	BCFJ	ACEGHIK	BF	ACDEFGHIJK	BCDFG	ABCEFGHIJK	ABCDEFGHIJK
14	BCDEFIJK	AH	BCDEFGIJK	FH	CG	ABCDEFGHIJK	ABCDEFGHIJK
15	ABCDEFGHIJK	C	ABDEFGHIJK	C	CFI	ABCDEFGHJK	ABCDEFGHIJ

**Table 2:** Dissimilarities obtained by IAMA among the TCM physicians.

Doctor	A	B	C	D	E	F	G	H	I	J	K
A	0	0.6029	0.7538	0.4705	0.5076	0.5846	0.6285	0.3076	0.4769	0.5384	0.5454
B	0.6029	0	0.84	0.5166	0.5	0.42	0.5254	0.6	0.6153	0.4615	0.4629
C	0.7538	0.84	0	0.7454	0.7826	0.8974	0.8333	0.7647	0.7391	0.7826	0.8297
C	0.4705	0.5166	0.7454	0	0.4909	0.4727	0.5483	0.5263	0.5178	0.3818	0.5614
D	0.5076	0.5	0.7826	0.4909	0	0.5319	0.5789	0.3725	0.7659	0.3913	0.4693
E	0.5846	0.42	0.8974	0.4727	0.5319	0	0.5660	0.5384	0.5319	0.5319	0.5833
F	0.6285	0.5254	0.8333	0.5483	0.5789	0.5660	0	0.6557	0.7192	0.6140	0.6206
G	0.3076	0.6	0.7647	0.5263	0.3725	0.5384	0.6557	0	0.5576	0.6078	0.6296
H	0.4769	0.6153	0.7391	0.5178	0.7659	0.5319	0.7192	0.5576	0	0.5957	0.6326
I	0.5384	0.4615	0.7826	0.3818	0.3913	0.5319	0.6140	0.6078	0.59574	0	0.6326
J	0.5454	0.4629	0.829787	0.5614	0.4693	0.5833	0.6206	0.6296	0.63265	0.6326	0

## Appendices

### A. IAMA Responses Proposed by Kupper and Hafner

Consider a study in which two equally trained raters, say raters A and B, independently examine each of *N* units. Let *A*_*i*_ denote the subset of attribute for the *i*th unit chosen by rater A, and let card(*A*_*i*_) = *a*_*i*_, 0 ≤ *a*_*i*_ ≤ *k*, denote the cardinality of set *A*_*i*_. The symbol $\bar{A}$ stands for the complement of set *A*. We may depict the data for the *i*th unit as follows.

Define the random variable

(A.1) Yi=card(Ai∩Bi)+card(A−i∩B−i)=(k−ai−bi+2Xi)

to be the number of attributes for the *i*th unit either chosen by both raters or not chosen by either rater. Define the following agreement proportion:

(A.2) $$\begin{array}{c} {\hat{\pi }}_{i}=\frac{Y_{i}}{k}, \end{array}$$

the overall concordance

(A.3) $$\begin{array}{c} \hat{\pi }=\frac{1}{N}\sum_{i=1}^{N}{\hat{\pi }}_{i}, \end{array}$$

and the chance-corrected concordance

(A.4) $$\begin{array}{c} {\hat{\pi }}_{AB}=\frac{\hat{\pi }-\pi_{0}}{1-\pi_{0}},\;\text{where}\;\pi_{0}=\frac{1}{Nk}\sum_{i=1}^{N}\min\;\left(a_{i},b_{i}\right). \end{array}$$

### B. MCD and IOA Differences

The mean character difference (MCD) and index of association (IOA) are popular distances used in MDS analysis. Let *x* = (*x*_1_,…, *x*_*n*_) and *y* = (*y*_1_,…, *y*_*n*_) be two vectors of attributes. The MCD distance is defined as

(B.1) $$\begin{array}{c} d\left(x,y\right)=\frac{1}{n}\sum_{i=1}^{n}\left|x_{i}-y_{i}\right|, \end{array}$$

and the IOA distance is defined by

(B.2) $$\begin{array}{c} d\left(x,y\right)=\frac{1}{2}\sum_{i=1}^{n}\left|\frac{x_{i}}{\sum_{j=1}^{n}x_{j}}-\frac{y_{i}}{\sum_{j=1}^{n}y_{j}}\right|. \end{array}$$
